# Multidimensional Profiles of Health Status: An Application of the Grade of Membership Model to the World Health Survey

**DOI:** 10.1371/journal.pone.0004426

**Published:** 2009-02-10

**Authors:** Alessandra Andreotti, Nadia Minicuci, Paul Kowal, Somnath Chatterji

**Affiliations:** 1 CNR- Institute of Neuroscience, Padova Section, Padova, Italy; 2 Multi-Country Studies Unit, Health Statistics and Informatics (HSI), World Health Organization, Geneva, Switzerland; CIET, Canada

## Abstract

**Background:**

The World Health Organization (WHO) conducted the World Health Survey (WHS) between 2002 and 2004 in 70 countries to provide cross-population comparable data on health, health-related outcomes and risk factors. The aim of this study was to apply *Grade of Membership* (GoM) modelling as a means to condense extensive health information from the WHS into a set of easily understandable health profiles and to assign the degree to which an individual belongs to each profile.

**Principal Findings:**

This paper described the application of the GoM models to summarize population health status using World Health Survey data. Grade of Membership analysis is a flexible, non-parametric, multivariate method, used to calculate health profiles from WHS self-reported health state and health conditions. The WHS dataset was divided into four country economic categories based on the World Bank economic groupings (high, upper-middle, lower-middle and low income economies) for separate GoM analysis. Three main health profiles were produced for each of the four areas: I. *Robust*; II. *Intermediate*; III. *Frail*; moreover population health, wealth and inequalities are defined for countries in each economic area as a means to put the health results into perspective.

**Conclusions:**

These analyses have provided a robust method to better understand health profiles and the components which can help to identify healthy and non-healthy individuals. The obtained profiles have described concrete levels of health and have clearly delineated characteristics of healthy and non-healthy respondents. The GoM results provided both a useable way of summarising complex individual health information and a selection of intermediate determinants which can be targeted for interventions to improve health. As populations' age, and with limited budgets for additional costs for health care and social services, applying the GoM methods may assist with identifying higher risk profiles for decision-making and resource allocations.

## Introduction

Currently, the concept of health as defined by the World Health Organization (WHO) is “a state of complete physical, mental and social well-being and not merely the absence of disease or infirmity” [1]. Taking this perspective, one moves beyond disease absence as defining health status to one that incorporates the complex perceptions about health and health conditions. Measuring the multidimensional character of perceived health status, and then using the results meaningfully, remains a challenge for policy and research purposes. Many of the available analytical techniques used to reduce variables, make assumptions about distributions or use summary variables in the calculations. The alternative technique, Grade of Membership (GoM) model, is presented in this paper.

GoM is a non-parametric method that identifies latent health profiles and the degree to which an individual fits these profiles. The GoM method has been applied in previous studies for depressive symptoms and personality disorders [2], [3], [4], older adult health status [5], [6], [7], [8], [9] and genetic health studies [10], [11]. A method that helps to define and predict transitions from robust health to frailty or the reverse, as well as identify pre-disability states would be helpful in planning for an ageing population [12], [13], [14], [15].

The WHO's World Health Survey (WHS) gather data to quantify population health status in 70 countries based on WHO's definition of health. The main aim of this study was to summarize, using the *Grade of Membership* model, the full set of health and health-related variables included in the WHS into a smaller set of meaningful health profiles [16]. In order to make these derived health profiles useful in helping to inform health policy, WHS data have been grouped in four economic areas according to the World Bank economic categories [17].

This paper is organized into three sections. First, a description of the data set is provided, which includes details of the survey design, socio-demographic characteristics of the sample and health data. Then the GoM procedure and results of the GoM analysis are described for each economic category. The final section summarizes the results.

## Materials and Methods

### Data

The WHS was conducted between 2002 and 2004 in 70 countries to establish levels of health and to develop methods to improve data comparability within and across countries [18], [19]. The principal aim of the WHS was to provide valid, reliable and comparable information about population health status. It used a common survey instrument in nationally representative populations for assessing, amongst other issues, the health of individuals in eight of the 22 explicit health domains, health system responsiveness and household health care expenditures.

A probability sampling design was employed in each country using multi-stage, stratified, random cluster samples. The population included all selected persons aged 18 years and older who lived in selected households. Most of the countries had nationally representative survey samples and each country decided which interview method to use: face-to-face interview, computer-assisted telephone interview (CATI) and/or computer-assisted personal interview (CAPI).

The WHS utilized two types of questionnaires: the *Household Questionnaire* (to describe the health, economic physical characteristics at the household level) and the *Individual questionnaire* (to describe the individual health status and well-being characteristics). In order to construct the final dataset, data was extracted from the WHS Individual Questionnaire. The overall dataset was then divided into four economic areas for the analyses based on the World Bank categories: high income, upper middle income, lower middle income and low income (Table 1).

**Table 1:** World Health Survey country groupings by World Bank economic categories.

High Income Countries	Upper-middle Income Countries	Lower-middle Income Countries	Low Income Countries
Australia	Croatia	Bosnia and Herzegovina	Bangladesh
Austria	Brazil	China	Burkina Faso
Belgium	Hungary	Congo	Chad
Czech Republic	Kazakhstan	Dominican Republic	Comoros
Denmark	Latvia	Ecuador	Côte d'Ivoire
Estonia	Malaysia	Georgia	Ethiopia
Finland	Mauritius	Guatemala	Ghana
France	Mexico	Morocco	India
Germany	Russian Federation	Namibia	Kenya
Greece	Slovakia	Paraguay	Lao People's Democratic Republic
Ireland	South Africa	Philippines	Malawi
Israel	Uruguay	Sri Lanka	Mali
Italy		Swaziland	Mauritania
Luxembourg		Tunisia	Myanmar
Netherlands		Ukraine	Nepal
Norway			Pakistan
Portugal			Senegal
Slovenia			Vietnam
Spain			Zambia
Sweden			Zimbabwe
United Arab Emirates			
United Kingdom			
22	12	15	20

The World Bank's uses gross national income (GNI) per capita as its main criterion for classifying economies. Based on its 2006 GNI per capita, every country's economy was classified as low income, middle income (subdivided into lower middle and upper middle), or high income. The four groups are defined as: low income, $905 or less; lower middle income, $906–$3595; upper middle income, $3596–$11,115; and high income, $11,116 or more.

### Physical and mental health

The dataset included self-reported diagnosis of three physical and one mental health condition (arthritis, angina pectoris, asthma and depression), self-reported difficulties in functioning in eight health domains (mobility, self-care, pain and discomfort, cognition, interpersonal relationships, vision, sleep and energy, and affect) plus one self-reported overall health question. Presence or absence of a diagnosed condition was based on self-report.

### Grade of Membership method

Grade of Membership (GoM) model [20] is a flexible, non-parametric, multivariate method, designed to identify health profiles. In our work we used the self-reported health state and health conditions in order to determine the latent profiles (pure types) of health and the degree to which individuals correspond to the identified profiles (grade of membership).

Briefly, as outlined in Manton et al [20], the GoM model assumes there are *K* fuzzy states (pure types) to be defined. The study population consists of *I* individuals with *J* categorical variables, where the *j*th variable has L*_j_* response levels. Each L*_j_* response is encoded as a binary variable *x_ijl_*, so that if *x_ijl_* = 1 then the *i*th individual has the *l*th response to the *j*th variable. A first coefficient, λ*_kjl_*, concerns the likelihood of a response *l* to the *j*th question by an individual belonging to the *k*th health pure type; the second entity, *g_ik_*, represents weights quantifying the grade of similarity of the health features of the *i*th individual with the characteristics of each *K* pure types, with the following constraints: 0≤λ*_kjl_*≤1, 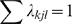, 0≤*g_ik_*≤1 and 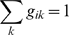.

By summing over all potential GoM health pure types: 
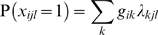
we obtain the probability that the *i*th individual responds *l* to question *j*.

Assuming independence of individual observations, the likelihood function for the GoM model is:
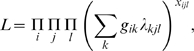
subject to: 0≤*g_ik_*≤1, 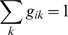 and 0≤λ*_kjl_*≤1, 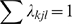.

We used the DSIGoM software [21] to estimate the GoM parameters. In particular the modified Newton-Raphson algorithm was employed, where the coefficients *g_ik_* and λ*_kjl_* are estimated simultaneously to maximize the likelihood function *L*. The parameters are estimated iteratively: the L function is maximized first with λ*_kjl_* fixed, producing an initial estimate of all *g_ik_*; then using the obtained *g_ik_* the L function is maximized to update the λ*_kjl_*, This process is repeated until convergence, where the parameters are such that within-group homogeneity is maximized and between-group homogeneity is minimized [20].

The optimal number of profiles is established by performing a likelihood ratio test on the change in explanatory power between *K* and *K*+1 model. This ratio is *χ* ^2^ distributed, with degrees of freedom equal to the difference in the number of parameters to be estimated between models [10].

### Grade of Membership application

Prior to analyzing data, it was necessary to define the external and the internal variables [10]. External variables do not affect the definitions of the pure types and included five socio-demographic variables (age, sex, marital status, education and employment); however, the association between the pure types and the external variables provides valuable information about the relationship between empirically-derived pure types and demographic characteristics. Turkey was not included in the dataset because the external variable marital status was not available.

The continuous age variable was recoded into three categories: younger adult (18–29 years); adult (30–59 years), and older adult (60 years and older). Marital status was recoded into four categories: never married, currently married/cohabiting, separated/divorced and widowed. Sex, education levels (highest level completed) and sector of current employment (governmental, non-governmental, self-employed, employer, homemaker, unemployed, student, retired and other) were the remaining external variables.

Internal variables included self-reported overall health (based on a five point scale: very good, good, moderate, bad or very bad), scores from the eight health domains (none, mild, moderate, severe or extreme/cannot do), and the set of four reported conditions (yes, no).

For each of the four country categories, the GoM analysis was applied with 2, 3 and 4 pure types to test for the optimal number of pure types. The GoM parameters estimation was derived using the DSIGoM software. Log likelihood ratio test indicated that three pure types provided the best description of the structure of the variables included in this analysis for each economic area. Each pure type was described by the values obtained for the λ coefficients. In general, λ*_kjl_*>0.50 was considered to be characteristic of a pure type being endorsed by more than 50% of individuals in that pure type. The lambda coefficients were produced for each of the external and internal variables. Additionally, the distribution of respondents' GoM scores (*g_ik_*) was generated for each pure type and country category. The crude prevalence estimates refer to the sum of individual membership in the *k*th pure type, divided by the total number of respondents,
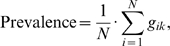
where *N* is the total number of respondents, and *g_ik_* is the GoM coefficient for the *i*th individual's degree of membership in the *k*th pure type [4].

In order to compare the prevalence rates across the four groups, age-standardized prevalence estimates were calculated. For each pure type and economic category, age-specific (younger adult 18–29, adult 30–59, and older adult 60+) prevalence ratios were computed. To calculate adjusted age-specific prevalence rates we used the direct standardization method with the WHO world standard population table [22].

## Results

### Socio-demographic characteristics

The final dataset contained 217,472 respondents from 69 countries. These countries were grouped into the four World Bank income categories for analysis. The socio-demographic characteristics of the respondents are provided in Table 2.

**Table 2:** Socio-demographic characteristics (%) by World Bank economic category.

Variables	HIGH INCOME	UPPER MIDDLE INCOME	LOWER MIDDLE INCOME	LOW INCOME	p-value
	(N = 26358)	(N = 51090)	(N = 58799)	(N = 81225)	(Χ^2^)
Sex					<0.0001
Female	50.60	52.94	51.02	49.49	
Male	49.40	47.06	48.98	50.51	
Age					<0.0001
18–19	3.57	5.88	5.77	8.09	
20–29	17.76	26.27	27.37	32.21	
30–39	18.84	21.68	20.91	21.77	
40–49	18.95	18.42	18.42	16.02	
50–59	15.91	12.34	12.25	10.99	
60–69	10.72	8.19	8.73	6.80	
70+	14.25	7.21	6.55	4.13	
Marital Status					<0.0001
Never Married	22.82	26.88	25.40	21.78	
Currently Married	54.12	48.72	58.22	69.98	
Separated	2.29	3.07	1.60	0.79	
Divorced	5.03	3.58	2.19	1.12	
Widowed	8.58	8.01	7.17	6.00	
Cohabiting	7.15	9.74	5.42	0.33	
Education					<0.0001
No formal schooling	2.44	7.01	9.73	40.09	
Less than primary school	5.88	7.82	9.76	13.30	
Primary school completed	16.66	21.77	21.14	19.71	
Secondary school completed	23.67	27.04	23.96	13.45	
High school(or equivalent) completed	27.48	24.31	19.67	7.39	
College/pre-university/University completed	16.68	10.34	15.18	5.09	
Post graduate degree completed	7.21	1.72	0.55	0.96	
Current job					<0.0001
Government employee	14.46	14.64	12.79	4.05	
Private sector employee	39.90	23.56	16.98	7.50	
Self-employed	6.12	19.60	19.54	45.80	
Employer	1.60	2.57	2.17	0.70	
Homemaker	10.08	10.25	20.84	27.14	
Looked but can't find a job	3.74	8.79	7.88	3.04	
Studies	7.49	3.64	5.40	4.38	
Retired	13.77	11.94	10.03	4.17	
Other	2.84	5.01	4.36	3.22	

Three country categories (high, upper-middle and lower-middle income) had more women than the other group (low income), although the subdivision between males and females is almost symmetric for every region. More young respondents (aged 20–29 years) were noted in the upper-middle, lower-middle and low income categories. The higher income categories had more old respondents than the other groups. The low income category had a higher percentage (8.1%) of the youngest respondents (aged 18–19 years), while the high income category had the highest percentage of older adults with 14.2% of individuals aged 70+ years. Respondents in the low income category were more likely to be currently married (70%) and had the highest levels of respondents with no formal education (40.1%). The high income category had the highest education rate. Current employment sector/issues differed by category: the two highest income categories (high and upper-middle income) had more respondents employed in the private sector (39.9% and 23.6% respectively). The lower-middle income category had more homemakers and self-employed (20.8% and 19.5%) while low income countries had higher levels of self-employed (45.8%). The category with the most retired respondents was the high income (13.8%). An association between all socio-demographic characteristics and the four economic country categories was found (p<0.0001).

### Physical and mental health data

The internal variables in the GoM analysis included self-reported physical and mental health data. Descriptive statistics of these variables for the four economic categories were provided in Table 3.

**Table 3:** Internal variables descriptive statistics (%) by World Bank economic category.

Variables	HIGH INCOME		UPPER MIDDLE INCOME	LOWER MIDDLE INCOME	LOW INCOME	p-value	
	(N = 26358)		(N = 51090)	(N = 58799)	(N = 81225)	(Χ^2^)	
Health Status						<0.0001	
Very Good		23.65	17.11	13.99	25.05		
Good		46.42	40.99	39.47	38.92		
Moderate		22.23	33.20	35.38	26.62		
Bad		6.66	7.18	9.23	8.06		
Very Bad		1.05	1.52	1.93	1.35		
Difficulty moving around						<0.0001	
None		75.72	72.71	61.12	65.05		
Mild		9.75	11.61	15.75	18.36		
Moderate		9.14	9.73	17.15	10.00		
Severe		4.48	4.94	5.18	5.39		
Extreme/Cannot do		0.91	1.02	0.81	1.20		
Difficulty in Self-Care						<0.0001	
None		89.56	87.96	79.73	78.63		
Mild		5.16	5.82	9.39	12.53		
Moderate		3.40	3.82	8.52	5.37		
Severe		1.41	1.79	1.81	2.52		
Extreme/Cannot do		0.47	0.61	0.55	0.96		
Pain and discomfort						<0.0001	
None		52.14	46.20	44.64	47.53		
Mild		24.55	23.80	25.06	27.38		
Moderate		14.97	18.47	19.24	14.10		
Severe		7.31	8.76	9.51	9.01		
Extreme		1.03	2.77	1.55	1.97		
Difficulty in concentration						<0.0001	
None		69.55	61.99	59.41	64.45		
Mild		17.98	19.30	19.82	19.43		
Moderate		9.36	11.76	15.12	9.76		
Severe		2.67	5.73	4.80	5.12		
Extreme/Cannot do		0.44	1.22	0.84	1.25		
Difficulty in personal relationships						<0.0001	
None		85.48	81.34	71.50	77.84		
Mild		8.55	9.99	14.63	13.23		
Moderate		4.22	5.82	11.21	5.29		
Severe		1.44	2.00	1.98	2.34		
Extreme/Cannot do		0.31	0.85	0.67	1.31		
Difficulty in seeing and recognizing persons						<0.0001	
None		83.83	78.33	73.95	79.65		
Mild		8.88	9.91	11.88	10.19		
Moderate		4.64	6.74	8.78	5.27		
Severe		1.96	3.60	4.30	3.50		
Extreme/Cannot do		0.70	1.41	1.08	1.39		
Sleeping disorders						<0.0001	
None		58.95	60.95	56.48	65.37		
Mild		19.47	17.34	20.46	18.12		
Moderate		14.15	12.63	15.18	9.59		
Severe		6.08	7.05	6.77	5.41		
Extreme		1.35	2.03	1.11	1.50		
Feeling sad or depressed						<0.0001	
None		64.06	53.89	54.20	59.57		
Mild		19.71	21.68	23.37	22.00		
Moderate		10.86	14.48	14.91	10.74		
Severe		4.41	7.20	6.28	5.99		
Extreme		0.95	2.75	1.24	1.70		
Diagnosis of Arthritis						<0.0001	
No		86.88	90.15	86.53	85.83		
Yes		13.12	9.85	13.47	14.17		
Diagnosis of Angina Pectoris						<0.0001	
No		95.17	92.56	91.91	93.48		
Yes		4.83	7.44	8.09	6.52		
Diagnosis of Asthma						<0.0001	
No		90.18	93.29	94.57	95.95		
Yes		9.82	6.71	5.43	4.05		
Diagnosis of Depression						<0.0001	
No		85.88	90.05	96.09	94.88		
Yes		14.12	9.95	3.91	5.12		

The majority of the respondents reported good or very good health status for the self-reported overall general health question, with the lower-middle income category having the highest percentage of respondents reporting a bad or very bad health status. In all the eight health domains (mobility, self-care, pain and discomfort, cognition, interpersonal relationships, vision, sleep and energy, and affect), the prevalence rates followed a positive trend. The majority of the respondents (more than 50%) reported no difficulties on any of the physical or mental health issues, with the exception of the domain “pain and discomfort”, where prevalence rates ranged from 44.6% of lower-middle income category to 52.1% of high income group. Finally, over 85% of respondents reported no diagnosed health conditions (arthritis, angina pectoris, asthma and depression). Among these conditions, it was noted that the low income category had the highest percentage (14.2%) of respondents with arthritis, the lower-middle income group had the highest percentage (8.1%) with angina pectoris, and the high income category had the highest percentage with asthma and depression (9.8% and 14.1%, respectively).

### GoM parameters and pure type estimation

Table 4 provides a summary of the pure types/health profiles by World Bank category. The components of pure types I (ROBUST) and II (INTERMEDIATE) are very similar across all the categories. Moving from type I to type II resulted in increasing difficulty in some health domains, with respondents more likely reporting “moderate difficulty” (INTERMEDIATE) instead of “no difficulty” (ROBUST) for the given health domain. The third health profile, FRAIL, was again a distinctly lower level of health based on difficulties with the health domains and presence of one or more of the health conditions.

**Table 4:** General characteristics of the internal variables by pure type and World Bank economic category (listing the predominant Lambda probability λ*_kjl_* by variable (for more details see the appendix)).

Profile (Pure type)	Variables	High Income	Upper Middle Income	Lower Middle Income	Low Income
I: ROBUST	SRH	Good	Good	Good	Good
	HS	None	None	None	None
	CC	None	None	None	None
II: INTERMEDIATE	SRH	Good	Moderate	Moderate	Moderate
	HS	Some	Some	Some	Some
	CC	None	None	None	None
III: FRAIL	SRH	Moderate	Moderate	Moderate	Moderate
	HS	More	More	More	More
	CC	Arthritis, Depression	Arthritis	None	Arthritis

* SRH = Self-reported overall general health (“In general, how would you rate your health today?”), HS = health state determined by level of difficulty with each of the eight health domains, CC = reported chronic conditions: arthritis, angina pectoris, asthma and/or depression.

Similarly, the lambda probability variables for each of the external variables by country category show discernable patterns for each of the health profiles (Table 5).

**Table 5:** General characteristics of the external variables by pure type and World Bank economic category (listing the predominant *Lambda* probability λ*_kjl_* by variable).

Profile (Pure type)	Variables	High Income	Upper-middle Income	Lower-middle Income	Low Income
I: ROBUST	Sex	Female/Male	Male	Male	Male
	Age group	Adult	Adult	Adult	Young/Adult
	Marital status	Married	Married	Married	Married
	Education	High	Intermediate	Low	None
	Current job	Private sector	Self-employed	Self-employed	Self-employed
II: INTERMEDIATE	Sex	Female	Female	Female	Female
	Age group	Adult	Adult	Adult	Adult
	Marital status	Married	Married	Married	Married
	Education	High	Intermediate	Low	None
	Current job	Private sector	Self-employed	Homemaker	Self-employed
III: FRAIL	Sex	Female	Female	Female	Female
	Age group	Older	Older	Older	Adult
	Marital status	Married	Married	Married	Married
	Education	Low	Low/Intermediate	None	None
	Current job	Retired	Retired	Homemaker	Self-employed

### High income economies

Table 6 shows the distribution of individual GoM coefficients (*g_ik_*) for the 26,358 high income respondents. Sixty-four percent (N = 16,940) had a high grade of membership (*g_ik_*>0.50) to pure type I. 25 percent of respondents (N = 6,648) belonged exclusively to one of the three pure types. Most respondents from this high income category (62.4%) belonged to the ROBUST profile (that is, pure type I).

**Table 6:** **High income countries:** Distribution of respondents' GoM scores (*g_ik_*) for each pure type (n = 26358).

GoM range*	I		II		III	
	*N*	%	*n*	%	*n*	%
0	2958	11.22	10958	41.57	12614	47.86
0.01–0.25	1800	6.83	4957	18.81	6209	23.56
0.26–0.50	4660	17.68	6045	22.93	3785	14.36
0.51–0.75	6009	22.80	3192	12.11	2229	8.46
0.76–0.99	5117	19.41	960	3.64	933	3.54
1	5814	22.06	246	0.93	588	2.23
Age-standardized prevalence (%)	**62.4**		**21.8**		**15.8**	

* GoM scores range from 0 (no membership in that health profile) to 1 (exclusive membership in that health profile).

Table 7 shows the exact breakdown of the lambda probability values for each pure type. Respondents belonging to pure type I were equally distributed between men and women (lambda equal to 49.8% and 50.2%, respectively), mainly adults (62.6%), married or cohabiting (63.4%), intermediate or higher educated persons (30.1% and 31.1%, respectively), and not government employed (37.8%). They reported good health status (55%), had no difficulties with physical and mental activities (100%) and none of the four health conditions (lambda equal to 100% and 94.9%).

**Table 7:** **High income countries:** *lambda* coefficients of external and internal variables for each pure type.

	Freq (%)	I	II	III
**External variables**				
Sex				
Female	57.56	**49.80**	**64.15**	**74.71**
Male	42.44	**50.20**	35.85	25.29
Age				
Young Adult (18–29)	17.21	23.30	15.06	1.26
Adult (30–59)	53.97	**62.57**	**52.34**	29.84
Old Adult (60+)	28.83	14.13	32.60	**68.90**
Marital status				
Never Married	20.57	25.98	18.27	5.96
Currently married/Cohabiting	59.98	**63.45**	**58.86**	**50.16**
Separated/Divorced	8.89	7.23	10.76	11.99
Widowed	10.55	3.34	12.11	31.89
Education				
No formal schooling	2.57	0.75	2.01	9.02
Less than primary school	5.68	2.30	5.15	17.04
Primary school completed	14.74	10.67	13.45	**29.16**
Secondary school completed	28.03	**30.07**	23.85	26.45
High school (or equivalent) completed	27.12	**31.13**	**27.89**	13.48
College/pre-university/University completed	17.64	19.70	23.60	4.12
Post graduate degree completed	4.22	5.37	4.05	0.72
Current job				
Government employee	14.03	17.48	15.37	2.15
No government employee	29.41	**37.84**	**28.51**	5.04
Self-employed	6.86	8.52	6.72	2.00
Employer	1.95	2.55	1.64	0.48
Homemaker	13.13	11.51	11.39	20.00
Looked but can't find a job	3.13	3.64	2.90	1.88
Studies	5.08	6.56	5.47	0.20
Retired	22.02	10.48	25.42	**52.92**
Other	4.38	1.43	2.57	15.33
**Internal variables**				
Health Status				
Very Good	25.00	45.02	0.00	0.00
Good	43.13	**54.98**	**60.59**	0.00
Moderate	24.38	0.00	39.41	**68.36**
Bad	6.23	0.00	0.00	26.30
Very Bad	1.26	0.00	0.00	5.34
Difficulty moving around				
None	71.87	**100.00**	46.99	0.00
Mild	12.28	0.00	**53.01**	0.00
Moderate	10.33	0.00	0.00	**65.21**
Severe	4.69	0.00	0.00	29.57
Extreme/Cannot do	0.83	0.00	0.00	5.22
Difficulty in Self-Care				
None	89.86	**100.00**	**100.00**	**34.00**
Mild	5.18	0.00	0.00	**33.71**
Moderate	3.40	0.00	0.00	22.10
Severe	1.24	0.00	0.00	8.05
Extreme/Cannot do	0.33	0.00	0.00	2.15
Pain and discomfort				
None	48.92	**100.00**	0.00	0.00
Mild	25.35	0.00	**100.00**	0.00
Moderate	16.81	0.00	0.00	**65.34**
Severe	7.82	0.00	0.00	30.38
Extreme	1.10	0.00	0.00	4.28
Difficulty in concentration				
None	68.32	**100.00**	0.00	0.00
Mild	19.56	0.00	**100.00**	0.00
Moderate	9.11	0.00	0.00	**75.12**
Severe	2.69	0.00	0.00	22.19
Extreme/Cannot do	0.33	0.00	0.00	2.69
Difficulty in personal relationships				
None	83.19	**100.00**	**67.20**	**37.15**
Mild	9.97	0.00	32.80	19.13
Moderate	4.69	0.00	0.00	29.95
Severe	1.74	0.00	0.00	11.13
Extreme/Cannot do	0.41	0.00	0.00	2.64
Difficulty in seeing and recognizing persons				
None	84.08	**100.00**	**71.85**	**46.58**
Mild	8.87	0.00	28.15	13.26
Moderate	4.50	0.00	0.00	25.60
Severe	1.97	0.00	0.00	11.19
Extreme/Cannot do	0.59	0.00	0.00	3.37
Sleeping disorders				
None	56.66	**100.00**	0.00	0.00
Mild	20.31	0.00	**81.32**	0.00
Moderate	14.53	0.00	18.68	**53.71**
Severe	7.17	0.00	0.00	39.06
Extreme	1.33	0.00	0.00	7.23
Feeling sad or depressed				
None	62.13	**100.00**	0.00	0.00
Mild	20.78	0.00	**100.00**	0.00
Moderate	11.42	0.00	0.00	**66.81**
Severe	4.73	0.00	0.00	27.67
Extreme	0.94	0.00	0.00	5.52
Diagnosis of Arthritis				
No	84.21	**100.00**	**83.64**	38.46
Yes	15.79	0.00	16.36	**61.54**
Diagnosis of Angina Pectoris				
No	93.87	**100.00**	**100.00**	**68.22**
Yes	6.13	0.00	0.00	31.78
Diagnosis of Asthma				
No	89.81	**94.92**	**86.03**	**77.79**
Yes	10.19	5.08	13.97	22.21
Diagnosis of Depression				
No	87.87	**100.00**	**100.00**	39.70
Yes	12.13	0.00	0.00	**60.30**

Individuals in pure type II differed from those in pure type I in that they were mainly female (64.1%) and had some difficulty with physical and mental activities, especially moving around (53%), pain and discomfort (100%), concentration (100%), sleeping (81.3%), feeling sad or depressed (100%).

Finally, respondents in pure type III were mainly female (74.7%), old (68.9%), married or cohabiting (50.2%), less educated (29.2%), and retired (52.9%). They reported moderate health status (68.4%) and had more difficulty with physical and mental activities, especially moving around (65.2%), pain and discomfort (65.3%), concentration (75.1%), sleeping (53.7%), feeling sad or depressed (66.8%). Moreover they reported having arthritis (61.5%) and depression (60.3%).

### Upper-middle income economies

Table 8 includes the *g_ik_* coefficients for the 51,090 respondents in the upper-middle income category. Almost 69 percent (N = 35,209) had a high grade of membership (*g_ik_*>0.50) for pure type I (ROBUST). Over 27 percent of respondents (N = 13,940) belonged exclusively to a single pure type. Most respondents from this region (62.3%) belong to the ROBUST health profile.

**Table 8:** **Upper-middle income countries:** Distribution of respondents' GoM scores (*g_ik_*) for each pure type (n = 51090).

GoM range*	I		II		III	
	*N*	%	*n*	%	*n*	%
0	5025	9.84	19597	38.36	27889	54.59
0.01–0.25	3103	6.07	11352	22.22	10866	21.27
0.26–0.50	7753	15.18	11844	23.18	6675	13.07
0.51–0.75	11861	23.22	6147	12.03	3301	6.46
0.76–0.99	10795	21.13	1712	3.35	1410	2.76
1	12553	24.57	438	0.86	949	1.86
Age-standardized prevalence (%)	**62.3**		**22.1**		**15.6**	

* GoM scores range from 0 (no membership in that health profile) to 1 (exclusive membership in that health profile).

Table 9 shows the lambda coefficient distributions of external and internal variables for each pure type. Respondents belonging to the pure type I were male (lambda equal to 63.8%), mainly adults (61.2%), married or cohabiting (65.5%), intermediate education levels (41.1%) and were not government employed (33.5%). They reported good health status (67.7%), had no difficulties with physical and mental activities (100%) and did not report any of the four health conditions (lambda equal to 100% and 98.2%).

**Table 9:** **Upper-middle income countries:** *lambda* coefficients of external and internal variables for each pure type.

	Freq (%)	I	II	III
**External variables**				
Sex				
Female	47.70	36.21	**60.04**	**76.28**
Male	52.30	**63.79**	39.96	23.72
Age				
Young Adult (18–29)	25.45	34.01	17.35	1.60
Adult (30–59)	59.85	**61.24**	**67.39**	43.14
Old Adult (60+)	14.70	4.75	15.26	**55.26**
Marital status				
Never Married	20.81	25.76	16.02	7.04
Currently married/Cohabiting	62.85	**65.53**	**65.25**	**47.93**
Separated/Divorced	8.12	6.41	10.02	12.49
Widowed	8.22	2.29	8.71	32.54
Education				
No formal schooling	3.57	1.26	3.58	13.02
Less than primary school	5.39	3.20	4.37	15.84
Primary school completed	16.42	14.79	14.95	**25.26**
Secondary school completed	35.69	**41.15**	**28.27**	**24.23**
High school (or equivalent) completed	25.98	29.34	**27.16**	10.49
College/pre-university/University completed	11.16	8.79	18.59	10.03
Post graduate degree completed	1.79	1.48	3.09	1.15
Current job				
Government employee	16.86	19.10	18.33	4.74
No government employee	28.72	**33.49**	**29.26**	6.90
Self-employed	26.09	29.74	23.14	14.54
Employer	1.89	2.03	2.27	0.70
Homemaker	8.28	6.88	10.70	10.75
Looked but can't find a job	3.87	3.37	4.28	5.40
Studies	2.11	2.58	2.13	0.04
Retired	9.54	1.70	7.98	**46.48**
Other	2.63	1.11	1.92	10.44
**Internal variables**				
Health Status				
Very Good	18.30	32.30	0.00	0.00
Good	45.65	**67.70**	29.56	0.00
Moderate	29.08	0.00	**70.44**	**62.71**
Bad	5.96	0.00	0.00	31.92
Very Bad	1.00	0.00	0.00	5.38
Difficulty moving around				
None	76.48	**100.00**	45.38	0.00
Mild	12.79	0.00	**54.62**	0.00
Moderate	7.00	0.00	0.00	**65.29**
Severe	3.13	0.00	0.00	29.18
Extreme/Cannot do	0.59	0.00	0.00	5.53
Difficulty in Self-Care				
None	90.45	**100.00**	**88.11**	**43.75**
Mild	5.58	0.00	11.89	23.66
Moderate	2.64	0.00	0.00	21.63
Severe	1.01	0.00	0.00	8.26
Extreme/Cannot do	0.33	0.00	0.00	2.70
Pain and discomfort				
None	53.18	**100.00**	0.00	0.00
Mild	24.39	0.00	**100.00**	0.00
Moderate	14.94	0.00	0.00	**66.59**
Severe	6.34	0.00	0.00	28.26
Extreme	1.16	0.00	0.00	5.16
Difficulty in concentration				
None	69.28	**100.00**	0.00	0.00
Mild	18.84	0.00	**100.00**	0.00
Moderate	8.09	0.00	0.00	**68.14**
Severe	3.26	0.00	0.00	27.49
Extreme/Cannot do	0.52	0.00	0.00	4.37
Difficulty in personal relationships				
None	84.12	**100.00**	**57.57**	**50.50**
Mild	10.57	0.00	42.43	11.10
Moderate	3.81	0.00	0.00	27.54
Severe	1.14	0.00	0.00	8.24
Extreme/Cannot do	0.36	0.00	0.00	2.62
Difficulty in seeing and recognizing persons				
None	79.70	**100.00**	**56.90**	29.94
Mild	11.23	0.00	43.10	8.31
Moderate	5.58	0.00	0.00	**37.96**
Severe	2.68	0.00	0.00	18.24
Extreme/Cannot do	0.81	0.00	0.00	5.54
Sleeping disorders				
None	65.62	**100.00**	0.00	0.00
Mild	18.80	0.00	**100.00**	0.00
Moderate	9.58	0.00	0.00	**61.48**
Severe	5.06	0.00	0.00	32.45
Extreme	0.95	0.00	0.00	6.07
Feeling sad or depressed				
None	61.80	**100.00**	0.00	0.00
Mild	22.38	0.00	**100.00**	0.00
Moderate	10.25	0.00	0.00	**64.80**
Severe	4.53	0.00	0.00	28.64
Extreme	1.04	0.00	0.00	6.56
Diagnosis of Arthritis				
No	91.38	**100.00**	**100.00**	47.36
Yes	8.62	0.00	0.00	**52.64**
Diagnosis of Angina Pectoris				
No	93.98	**100.00**	**100.00**	**60.47**
Yes	6.02	0.00	0.00	39.53
Diagnosis of Asthma				
No	95.06	**98.25**	**93.99**	**83.57**
Yes	4.94	1.75	6.01	16.43
Diagnosis of Depression				
No	94.10	**100.00**	**100.00**	**63.75**
Yes	5.90	0.00	0.00	36.25

Individuals in pure type II were mainly female (60%), reported moderate health status (70.4%) and had some difficulty with physical and mental activities, especially moving around (54.6%), pain and discomfort (100%), concentration (100%), sleeping (100%), feeling sad or depressed (100%).

Respondents in pure type III were mainly female (76.3%), old (55.3%), married or cohabiting (47.9%), intermediate or lower education levels (24.2% and 25.3%, respectively), and retired (46.5%). They reported moderate health status (62.7%) and had more difficulty with physical and mental activities, especially moving around (65.3%), pain and discomfort (66.6%), concentration (68.1%), sleeping (61.5%), feeling sad or depressed (64.8%) and had arthritis (52.6%).

### Lower-middle income economies

Table 10 presents the distribution of *g_ik_* scores for the 58,799 respondents from the lower-middle income category. Almost 64 percent of respondents (N = 37,537) had a high grade of membership (*g_ik_*>0.50) for pure type I. Twenty-two percent (N = 13,019) were exclusively in one pure type and 58.2 percent were in the ROBUST profile.

**Table 10:** **Lower-middle Income countries:** Distribution of respondents' GoM scores (gik) for each pure type (n = 58799).

GoM range*	I		II		III	
	*n*	%	*n*	%	*n*	%
0	6601	11.23	22813	38.80	25817	43.91
0.01–0.25	4434	7.54	13307	22.63	11469	19.51
0.26–0.50	10227	17.39	15642	26.60	10894	18.53
0.51–0.75	12412	21.11	5355	9.11	6080	10.34
0.76–0.99	13841	23.54	1421	2.42	3065	5.21
1	11284	19.19	261	0.44	1474	2.51
Age-standardized prevalence (%)	**58.2**		**20.0**		**21.8**	

* GoM scores range from 0 (no membership in that health profile) to 1 (exclusive membership in that health profile).

Table 11 shows the exact breakdown of the lambda probability values for the external and internal variables in each pure type. Respondents that belonged to the pure type I were male (lambda equal to 53.7%), mainly adults (56.4%), married or cohabiting (64.2%), lower education levels (26.3%), and not government employed (27.1%). They reported good health status (61.7%), no difficulties with physical or mental activities (lambda equal to 100% and 98.2%) and did not report any of the four health conditions (lambda equal to 100% and 98%).

**Table 11:** **Lower-middle income countries:** *lambda* coefficients of external and internal variables for each pure type.

	Freq (%)	I	II	III
**External variables**				
Sex				
Female	55.66	46.29	**66.87**	**70.35**
Male	44.34	**53.71**	33.13	29.65
Age				
Young Adult (18–29)	28.38	39.72	15.64	8.71
Adult (30–59)	55.70	**56.42**	**62.18**	**47.56**
Old Adult (60+)	15.92	3.86	22.18	43.73
Marital status				
Never Married	22.18	30.44	10.27	10.76
Currently married/Cohabiting	64.85	**64.20**	**73.56**	**58.46**
Separated/Divorced	4.73	3.53	5.94	6.89
Widowed	8.24	1.84	10.22	23.89
Education				
No formal schooling	15.66	10.36	10.66	**34.38**
Less than primary school	16.76	15.32	15.54	21.70
Primary school completed	25.29	**26.31**	**26.21**	21.75
Secondary school completed	19.99	22.53	23.52	10.01
High school (or equivalent) completed	11.90	14.00	11.03	7.13
College/pre-university/University completed	9.63	10.34	12.70	4.89
Post graduate degree completed	0.76	1.15	0.34	0.13
Current job				
Government employee	8.42	9.93	9.75	2.90
No government employee	15.29	20.39	10.05	5.94
Self-employed	25.25	**27.07**	25.84	19.59
Employer	1.50	1.29	2.27	1.35
Homemaker	25.10	21.68	**31.55**	**28.57**
Looked but can't find a job	8.04	10.19	4.08	5.79
Studies	4.10	5.28	0.89	3.85
Retired	7.74	1.46	12.19	21.13
Other	4.56	2.71	3.38	10.87
**Internal variables**				
Health Status				
Very Good	18.99	38.29	0.00	0.00
Good	37.99	**61.71**	28.75	0.00
Moderate	32.28	0.00	**71.25**	**56.61**
Bad	8.91	0.00	0.00	36.00
Very Bad	1.83	0.00	0.00	7.38
Difficulty moving around				
None	67.66	**100.00**	0.00	0.00
Mild	13.55	0.00	**100.00**	0.00
Moderate	13.18	0.00	0.00	**70.13**
Severe	4.87	0.00	0.00	25.91
Extreme/Cannot do	0.74	0.00	0.00	3.96
Difficulty in Self-Care				
None	83.02	**100.00**	**71.84**	31.52
Mild	7.96	0.00	28.16	15.99
Moderate	6.60	0.00	0.00	**38.38**
Severe	1.92	0.00	0.00	11.16
Extreme/Cannot do	0.51	0.00	0.00	2.94
Pain and discomfort				
None	46.79	**100.00**	0.00	0.00
Mild	23.88	0.00	**100.00**	0.00
Moderate	18.19	0.00	0.00	**62.00**
Severe	9.78	0.00	0.00	33.33
Extreme	1.37	0.00	0.00	4.67
Difficulty in concentration				
None	60.63	**100.00**	0.00	0.00
Mild	19.08	0.00	**100.00**	0.00
Moderate	13.81	0.00	0.00	**68.05**
Severe	5.64	0.00	0.00	27.78
Extreme/Cannot do	0.85	0.00	0.00	4.17
Difficulty in personal relationships				
None	77.71	**98.20**	43.30	42.94
Mild	10.67	0.00	**56.70**	0.00
Moderate	8.08	0.00	0.00	**43.93**
Severe	1.82	0.00	0.00	9.90
Extreme/Cannot do	1.72	1.80	0.00	3.23
Difficulty in seeing and recognizing persons				
None	74.54	**100.00**	44.44	28.58
Mild	11.01	0.00	**55.56**	0.00
Moderate	8.62	0.00	0.00	**42.61**
Severe	4.71	0.00	0.00	23.25
Extreme/Cannot do	1.13	0.00	0.00	5.56
Sleeping disorders				
None	60.03	**100.00**	0.00	0.00
Mild	18.41	0.00	**100.00**	0.00
Moderate	13.77	0.00	0.00	**63.85**
Severe	6.90	0.00	0.00	32.02
Extreme	0.89	0.00	0.00	4.13
Feeling sad or depressed				
None	56.46	**100.00**	0.00	0.00
Mild	21.22	0.00	**100.00**	0.00
Moderate	14.05	0.00	0.00	**62.96**
Severe	6.94	0.00	0.00	31.11
Extreme	1.32	0.00	0.00	5.93
Diagnosis of Arthritis				
No	86.78	**100.00**	**81.58**	**57.11**
Yes	13.22	0.00	18.42	42.89
Diagnosis of Angina Pectoris				
No	93.49	**100.00**	**100.00**	**70.99**
Yes	6.51	0.00	0.00	29.01
Diagnosis of Asthma				
No	95.00	**98.04**	**94.87**	**86.84**
Yes	5.00	1.96	5.13	13.16
Diagnosis of Depression				
No	95.07	**100.00**	**100.00**	**77.76**
Yes	4.93	0.00	0.00	22.24

Individuals in pure type II were mainly female (66.9%), homemakers (31.5%), reporting moderate health status (71.2%) and with some difficulty with physical and mental activities, especially moving around (100%), pain and discomfort (100%), concentration (100%), personal relationships (56.7%), seeing and recognizing persons (55.6%), sleeping (100%), feeling sad or depressed (100%).

Finally, respondents in pure type III were mainly female (70.3%), adults (47.6%), married or cohabiting (58.5%), not educated (34.4%), and homemakers (28.6%). They reported moderate health status (56.6%) and had more difficulty with physical and mental activities, especially moving around (70.1%), pain and discomfort (62%), concentration (68%), personal relationships (43.9%), seeing and recognizing persons (42.6%), sleeping (63.8%), feeling sad or depressed (63%); but did not report any of the four conditions.

### Low income economies

Table 12 shows the distribution of *g_ik_* scores for the 81,225 low income category respondents. Sixty-five percent (N = 53,151) of respondents had a high grade of membership (*g_ik_*>0.50) in pure type I (ROBUST) and over 25 percent (N = 20,667) of respondents were exclusively in one pure type. Overall, 58 percent of low income respondents belonged to the ROBUST profile (pure type I).

**Table 12:** **Low income countries**: Distribution of respondents' GoM scores (*g_ik_*) for each pure type (n = 81225).

GoM range*	I		II		III	
	*n*	%	*n*	%	*n*	%
0	9103	11.21	31132	38.33	39401	48.51
0.01–0.25	5793	7.13	17357	21.37	19195	23.63
0.26–0.50	13178	16.22	19777	24.35	11777	14.50
0.51–0.75	15885	19.56	9311	11.46	6144	7.56
0.76–0.99	19246	23.69	2766	3.41	2943	3.62
1	18020	22.19	882	1.09	1765	2.17
Age-standardized prevalence (%)	**58.4**		**22.4**		**19.2**	

* GoM scores range from 0 (no membership in that health profile) to 1 (exclusive membership in that health profile).

Table 13 shows the lambda coefficient distributions of external and internal variables for each pure type. Respondents belonging to pure type I were male (lambda equal to 53.4%), young or adults (lambda equal to 47.8% and 50%, respectively), married or cohabiting (70.7%), not educated (30.2%), and self-employed (52.1%). They reported good health status (52.1%), no difficulties with physical and mental activities (100%) and none of the four health conditions (100%).

**Table 13:** **Low income countries**: *lambda* coefficients of external and internal variables for each pure type.

	Freq (%)	I	II	III
**External variables**				
Sex				
Female	53.28	46.63	**60.14**	**67.47**
Male	46.72	**53.37**	39.86	32.53
Age				
Young Adult (18–29)	34.79	**47.81**	19.32	8.54
Adult (30–59)	53.03	**49.76**	**63.18**	**51.55**
Old Adult (60+)	12.19	2.43	17.51	39.92
Marital status				
Never Married	16.33	23.31	8.00	2.85
Currently married/Cohabiting	71.06	**70.75**	**76.63**	**65.19**
Separated/Divorced	4.25	3.46	4.51	6.67
Widowed	8.35	2.49	10.86	25.30
Education				
No formal schooling	41.55	**30.21**	**53.88**	**65.84**
Less than primary school	17.06	16.93	18.44	15.80
Primary school completed	20.46	24.89	14.73	12.11
Secondary school completed	11.53	15.07	7.86	3.75
High school (or equivalent) completed	4.93	6.77	2.69	1.29
College/pre-university/University completed	3.70	5.17	1.91	0.82
Post graduate degree completed	0.76	0.96	0.49	0.40
Current job				
Government employee	4.36	5.73	2.90	1.35
No government employee	6.77	8.71	4.13	3.23
Self-employed	48.97	**52.12**	**50.76**	**35.35**
Employer	0.93	0.86	1.19	0.83
Homemaker	22.77	18.96	29.74	27.38
Looked but can't find a job	4.23	5.52	1.80	2.76
Studies	3.45	5.15	1.16	0.33
Retired	4.68	0.68	5.74	17.60
Other	3.84	2.25	2.57	11.17
**Internal variables**				
Health Status				
Very Good	26.29	47.86	0.00	0.00
Good	39.75	**52.14**	43.27	0.00
Moderate	25.58	0.00	**56.73**	**56.81**
Bad	7.36	0.00	0.00	37.94
Very Bad	1.02	0.00	0.00	5.25
Difficulty moving around				
None	67.34	**100.00**	0.00	0.00
Mild	16.80	0.00	**100.00**	0.00
Moderate	9.59	0.00	0.00	**60.45**
Severe	5.35	0.00	0.00	33.73
Extreme/Cannot do	0.92	0.00	0.00	5.81
Difficulty in Self-Care				
None	79.73	**100.00**	**58.98**	0.00
Mild	11.70	0.00	41.02	26.54
Moderate	5.42	0.00	0.00	**46.50**
Severe	2.45	0.00	0.00	20.99
Extreme/Cannot do	0.70	0.00	0.00	5.96
Pain and discomfort				
None	48.54	**100.00**	0.00	0.00
Mild	26.93	0.00	**100.00**	0.00
Moderate	14.68	0.00	0.00	**59.85**
Severe	8.56	0.00	0.00	34.90
Extreme	1.29	0.00	0.00	5.25
Difficulty in concentration				
None	63.22	**100.00**	0.00	0.00
Mild	20.64	0.00	**100.00**	0.00
Moderate	10.32	0.00	0.00	**63.94**
Severe	4.98	0.00	0.00	30.82
Extreme/Cannot do	0.85	0.00	0.00	5.24
Difficulty in personal relationships				
None	77.89	**100.00**	45.99	28.32
Mild	13.01	0.00	**54.01**	11.46
Moderate	5.59	0.00	0.00	**37.00**
Severe	2.52	0.00	0.00	16.67
Extreme/Cannot do	0.99	0.00	0.00	6.55
Difficulty in seeing and recognizing persons				
None	78.08	**100.00**	**51.54**	29.70
Mild	10.46	0.00	48.46	0.00
Moderate	6.38	0.00	0.00	**39.12**
Severe	3.90	0.00	0.00	23.91
Extreme/Cannot do	1.19	0.00	0.00	7.27
Sleeping disorders				
None	63.53	**100.00**	0.00	0.00
Mild	19.09	0.00	**100.00**	0.00
Moderate	10.31	0.00	0.00	**59.35**
Severe	5.98	0.00	0.00	34.45
Extreme	1.08	0.00	0.00	6.20
Feeling sad or depressed				
None	56.87	**100.00**	0.00	0.00
Mild	23.16	0.00	**100.00**	0.00
Moderate	11.49	0.00	0.00	**57.52**
Severe	7.05	0.00	0.00	35.29
Extreme	1.44	0.00	0.00	7.19
Diagnosis of Arthritis				
No	85.40	**100.00**	**79.11**	48.20
Yes	14.60	0.00	20.89	**51.80**
Diagnosis of Angina Pectoris				
No	92.56	**100.00**	**93.81**	**67.84**
Yes	7.44	0.00	6.19	32.16
Diagnosis of Asthma				
No	95.85	**100.00**	**95.20**	**82.95**
Yes	4.15	0.00	4.80	17.05
Diagnosis of Depression				
No	93.69	**100.00**	**100.00**	**67.74**
Yes	6.31	0.00	0.00	32.26

Respondents in pure type II were mainly female (60.1%), reported moderate health status (56.7%) and had some difficulty with physical and mental activities, especially moving around (100%), pain and discomfort (100%), concentration (100%), personal relationships (54%), sleeping (100%), feeling sad or depressed (100%).

Finally, respondents in pure type III were mainly female (64.5%), adults (51.5%), married or cohabiting (65.2%), not educated (65.8%), and self-employed (35.3%). They had moderate health status (56.8%), more difficulty with physical and mental activities, especially moving around (60.4%), self-care (46.5%), pain and discomfort (59.8%), concentration (63.9%), sleeping (59.3%), feeling sad or depressed (57.5%), and arthritis (51.8%).

Age-standardized prevalence ratios of pure type I by economic category indicate similarity between the high and upper-middle income countries (both over 62%) and the lower-middle and low income countries (both less than 59%). Likewise, the two higher income categories had less than 16% membership in the FRAIL pure type whilst the lower-middle and low income countries had higher rates (21.8% and 19.2%, respectively).

## Discussion

This paper described the application of the Grade of Membership models to summarize population health status using World Health Survey data. The GoM model provided a meaningful method to reduce and summarize health variables from health surveys.

A number of techniques have previously been applied to WHS data to summarize and report on health status [18]. Comparing health results using Ustun's method to the GoM results indicated good face validity, with similar response patterns. Establishing comparable levels of health for different populations is extremely useful, but in addition to this, GoM provides a discrete set of profiles which are possibly easier to interpret and use for decision-making. The three health profiles for higher to lower income countries are digestible and realistic groupings of functioning and well-being. If universal health coverage were to be rolled out or expanded for the older population in a country, a policy maker might choose a stepped strategy starting with characteristics common in the frail profile. For example, this might include improving identification and treatments for selected comorbidities like depression and arthritis, along with items that have potential to improve functioning (like addressing pain or sleep problems) to allow for ageing (well) in place.

The GoM procedure differs from other classification methods, like Factor Analysis, which use indicators to calculate latent continuous variables that represent one-dimensional constructs. Factor Analysis results derive the parameter values from normally distributed data, whereas, the GoM model is a non-parametric method where identification of parameters does not rely on any distributional assumptions. Estimation of factor scores in Factor Analysis supports on distributional assumptions relating to the factor loadings [7]. GoM parameters are estimated in an iterative method: firstly, the likelihood function is maximized with λ*_kjl_* fixed, giving a first estimate of all *g_ik_*, then, fixing *g_ik_*, the likelihood is maximized to update the λ*_kjl_*, which is repeated until convergence.

Grade of Membership modelling shares similarities with other data reduction methods, such as Factor Analysis, Principal Component Analysis and Cluster Analysis. However, in the GoM model, all parameters are simultaneously identified, while, individual parameters in Factor Analysis and Principal Components methods are usually calculated using summary variables derived from within the dataset [7].

Additionally, in contrast to the Factor Analysis and Principal Component methods, GoM is a classification methodology where respondents are allocated to discrete and meaningful groups based on their grade of membership profile. Unlike other classification methodologies (such as Cluster Analysis), GoM does not generate groups of similar entities but considers individual heterogeneity [7]. GoM was, therefore, well suited for the planned analysis.

Grade of Membership analysis has been previously used to summarize health data from surveys for depressive symptoms and personality disorders, older people health status and genetic studies of health. Woodbury et al. [11] employed GoM analysis in a clinical setting to determine if the DSM-III-R personality disorder diagnostic criteria cluster into recognizable disorders. Four pure types provided the most satisfactory solution to the data. Portrait et al. [7] analyzed Longitudinal Aging Study Amsterdam data and identified six profiles to characterize health. Finally, Manton et al. [10] identified five profiles within the 1999 National Long Term Care Survey data, a national longitudinal survey based upon a list sample of US Medicare enrollers aged 65 years and above, which was used to demonstrate the compression of morbidity in the United States.

In this study, the GOM model produced three pure types (health profiles) for each economic category. Each health profile described unique facets of physical and mental health (internal GoM model variables) plus differences in socio-demographic characteristics (external GoM model variables) with a clear economic gradient (lower education and employment sector) when moving from high to low economic categories within each profile. Type I (ROBUST) and Type II (INTERMEDIATE) health profiles were more similar in both external and internal variables, as well as World Bank economic category, when compared to the Type III (FRAIL) profile.

- The *robust* profile respondents reported good health, none of the four health conditions and no difficulties with any of the physical and mental issues comprising the eight health domains.
- The *intermediate* profile respondents reported good or moderate health with increasing difficulties with some health domains but no reported health conditions.
- The *frail* profile clearly differed by external variables (older, more widowed, more retired or homemakers), internal variables (more difficulties with more of the eight health domains plus more likely to have a diagnosis of at least one of the health conditions) and economic category (the two lower economic categories had significantly higher rates of membership in the frail profile).

All four economic categories had somewhat similar robust and intermediate health profiles (pure types I and II). The two higher economic categories had more respondents in the robust pure type (greater than 64%) than the lower economic categories (less than 59%). Likewise, the two lower economic categories had more respondents in the frail pure types, (21.8% and 19.2%, respectively), with similar rates of membership in the intermediate profile across all four economic categories. The frail profile types may provide a logical focus for attention at all levels of country wealth, with policies targeted, for example, at older widowed women with mobility, sleep and cognition problems.

These analyses have provided a robust method to better understand health status and the components which can help to identify healthy and non-healthy individuals. Three profiles, *robust*, *intermediate* and *frail*, were obtained for respondents in each of the four economic categories. These profiles have described concrete levels of health as well as clearly delineating characteristics of healthy and non-healthy respondents. Areas for specific consideration include difficulties with sleep, mobility and depression, largely regardless of presence of specific health conditions or country of residence. The GoM results provided both a useable summary health measure and a selection of intermediate determinants which can be targeted for interventions to improve health. With limited health budgets, these results can help to make decisions about where health gains can be achieved. GoM would help to define specific characteristics within groups of individuals that can be targeted by health promotion efforts. As an example, specific health policy targets could address unmet need in a sub-population that encompass components of the frail profile. This could include a public health education campaign for health care professionals to look more closely at older married women reporting moderate health and problems with pain, sleep. It's more likely that they would also have comorbidities, such as arthritis and depression, to treat and may be undertreated. Treatment of these types of individuals could be part of a more comprehensive package to address well-being at older ages.

In future, we plan to investigate the transitions between health profiles, both improving and declining health, as well as the impact of the health-wealth relationship on shifts between profiles. We will additionally, look at the use of frailty definitions and profiles across different settings and the impact on disability assessments. These will provide the basis to inform policy about aging populations and measures to redress the determinants of more vulnerable health profiles. With a view to make results more cross-nationally comparable, vignette adjustments would improve ability to differentiate and correct for any reporting bias across countries and categories. This adjustment would also likely show more dramatic differences in health for respondents in lower income countries.
